# Objective analysis of the effectiveness of facial massage using breakthrough computed tomographic technology: A preliminary pilot study

**DOI:** 10.1111/srt.13152

**Published:** 2022-04-13

**Authors:** Itsuko Okuda, Mizuho Takeda, Masahiro Taira, Toyoaki Kobayashi, Ken Inomata, Naoki Yoshioka

**Affiliations:** ^1^ Department of Diagnostic Radiology International University of Health and Welfare (IUHW) Mita Hospital Tokyo Japan; ^2^ Department of Clinical Anatomy Tokyo Medical and Dental University (TMDU) Tokyo Japan; ^3^ Aging Imaging Laboratory Inc. Tokyo Japan; ^4^ Research Laboratories KOSÉ Corporation Tokyo Japan; ^5^ Department of Radiology International University of Health and Welfare (IUHW) Chiba Japan

**Keywords:** anti‐aging, buccal fat, cheek, facial massage, gravity, malar top, multidetector‐row computed tomography, spiral computed tomography, superficial musculoaponeurotic system

## Abstract

**Background:**

Facial massage is empirically known to be associated with morphological changes, such as improvements in facial sagging. However, quantified objective evaluations of massage‐induced changes have not been performed to date. This preliminary pilot study aimed to verify the effectiveness of facial massages by using breakthrough computed tomographic technology.

**Materials and methods:**

Five healthy adult volunteers (three women and two men; age, 29–37 years) were enrolled, and computed tomography (CT) examinations using a 320 detectors‐spiral CT system known as 320‐multidetector‐row CT (MDCT) were performed before and after facial massages. Each participant performed a self‐massage twice daily for 2 weeks. Massage‐induced changes in the cheeks and the superficial musculoaponeurotic system (SMAS) were analyzed by two radiologists on a workstation with a high‐accuracy imaging analysis system.

**Results:**

After facial massage, the malar top became thinner by −0.8% ± 0.45% and shifted cranially and horizontally over a distance of 3.9 ± 1.94 mm. The SMAS‐height, defined as the highest vertical distance of the SMAS, increased by 2.6% ± 2.6%. The change rate in cheek thickness and SMAS‐height showed a significant correlation (*r* = −0.63; *P* < 0.05). These changes were attributed to the lifting and tightening effects of facial massage.

**Conclusion:**

We conducted a detailed analysis of the effects of facial massages by using the breakthrough CT technology. Our results provide useful information for beauty treatments and could contribute to the collection of objective scientific evidence for facial massages.

## 1 INTRODUCTION

Interest in anti‐aging therapies and beauty treatments has been increasing in aging societies. In addition to providing mental satisfaction, relaxation, and improvements in skin texture,^1^, ^2^, ^3^, ^4^ beauty treatments such as facial massages also result in morphological changes in the face, such as improvements in facial sagging and lifting effects on the cheeks.^5^, ^6^ The soft tissues, including the superficial musculoaponeurotic system (SMAS) and subcutaneous adipose tissue,^7^, ^8^ are distributed subcutaneously, and facial massage may change their morphology.

To date, the effectiveness of facial massage has been mainly evaluated by subjective assessments such as visual methods and photographic comparisons. However, techniques based on stereo‐image correlation^5^ and computed tomography (CT)^6^ have been recently proposed for objective evaluation of the effectiveness of facial massage. Nevertheless, these evaluations were limited to changes in the surface morphology of the face, and they did not clarify the effects of facial massage on subcutaneous structures. Therefore, verifying the effect of facial massage, which has been discussed empirically, using an objective method was considered a highly noteworthy method.

Highly accurate and detailed three‐dimensional (3D) CT images can be constructed using advanced spiral CT known as the multidetector‐row CT (MDCT) technique for capturing high‐resolution images and a workstation for processing and analyzing a large volume of images.^7^, ^8^, ^9^ Moreover, spiral CT examination is a highly objective examination. The spiral CT imaging data obtained using this approach contain a large amount of anatomical information, which can be used to construct 3D images of the surface and subcutaneous structures of the face. In addition, multiplanar reformatted images, such as axial, coronal, and sagittal images, can be useful for understanding the anatomical relationship between morphological changes of the face and subcutaneous structures.

Therefore, this preliminary pilot study aimed to analyze the changes in the cheek state and SMAS following facial massage and thereby objectively determine the effectiveness of facial massages by using the breakthrough CT technology.

## 2 MATERIALS AND METHODS

### 2.1 Participants

This prospective study was approved by the institutional review board at the Mita Hospital of the International University of Health and Welfare (No. 5‐19‐40). Written informed consent was obtained from all the participants. Five healthy volunteers (three women and two men) without lesions that would influence the superficial facial structures were enrolled in the present study. The mean (± standard deviation) age of these participants was 33.8 ± 3.56 (range, 29–37) years.

**FIGURE 1:**
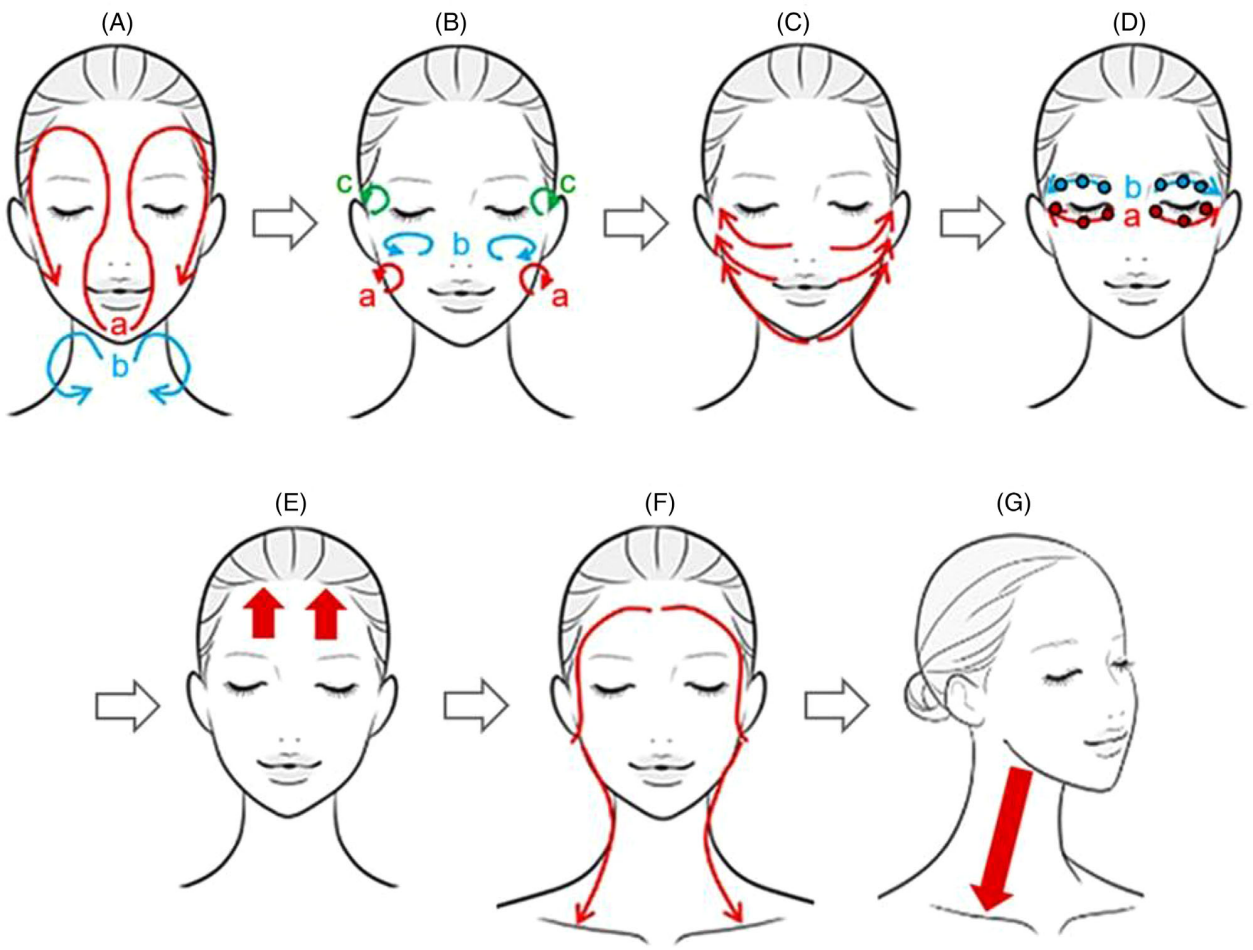
Self‐facial massage procedure. The self‐facial massage was done with the participants’ own hands to the face, using the following steps. (A) Put the milky lotion on both hands, and spread it over the face in a circular motion as shown in the red curved arrows (a). Next, spread it from the facial contour to the neck as shown in the blue curved arrows (b). (B) Place the dorsal parts of the middle phalanges of the four fingers (from second to fifth fingers) of both the clenched hands on the face, and relax in a small circular motion three times following the three areas; the buccal area (a), zygomatic area (b), and temporal area (c). (C) Place the thumbs under the chin, and the medial surface of the index fingers around the nose and mouth. Then, slide the fingers up to the temples three times as shown in the red arrows. (D) Place the fingertips of the middle and ring fingers on the lower eyelids, and slide these fingertips along the orbital edge from the inner to the outer corners three times (a). Perform the same procedure to the upper eyelids three times (b). After that, apply mild pressure to the three points (black open circles) of the lower and upper eyelids with the fingertips of the middle fingers. (E) Place the palm of the hand closely to the forehead, and pull the hand up toward the hairline. Do this three times on each side of the forehead as shown by the red arrows. (F) Place the second to the fifth fingers of both hands on the forehead, and slide these fingers from the forehead to the temples, in front of the ears, under the ears, and behind the ears, down to the collarbone. Do this three times as indicated by the red curved arrows. (G) With one hand, stroke the neck downward from below the jaw three times on each side of the neck

All five participants underwent CT examinations at the beginning of the study. Subsequently, they performed self‐facial massage in accordance with a specified method for approximately 90 s (Figure 1). The massages were performed using the same cosmetic emulsion daily in the morning and evening for 2 weeks. Self‐massage is a way to alleviate muscle stiffness and to pull up the cheeks using the fingers, based on attention to the lymphatic flow. Two weeks after they started performing self‐massages, all participants underwent a final massage by a professional technician using the same method as that used for the self‐facial massage but for double the duration. Subsequently, all five participants underwent a second CT examination.

### 2.2 Image acquisition

All the CT examinations were conducted using a spiral CT having 320 detectors known as the 320‐MDCT scanner (Aquilion ONE; Canon Medical Systems, Tochigi, Japan) with the following parameters: tube voltage, 120 kVp; tube current, 130–180 mA; exposure time, 1.0 s; and slice thickness, 0.5 mm. Whole‐face scans were obtained for each participant in the coronal positions of the face (C‐Fs). All CT imaging data were transferred to a workstation (ZioCube, Ziosoft Inc., Tokyo, Japan), and 3DCT images, including reconstituted CT images, were created.

### 2.3 Analysis of computed tomography images

Using the detailed CT imaging data of the C‐Fs, facial 3DCT and reconstructed CT images pre‐ and post‐facial massage were created on the workstation. Simultaneously, reconstructed CT images for analyzing the effectiveness of facial massage were created using the stationary facial bone as the landmark to allow accurate comparison of pre‐ and post‐massage images. CT images were analyzed by two radiologists (Itsuko Okuda and Naoki Yoshioka), each with more than 25 years of experience in consultation.

The following items were measured: (1) cheek thickness, (2) shifts in the location of the malar top, and (3) SMAS measurement. Measurements for each item were obtained three times using a digital caliper available on the workstation, and the average values were calculated.

#### 2.3.1 Cheek thickness

**FIGURE 2:**
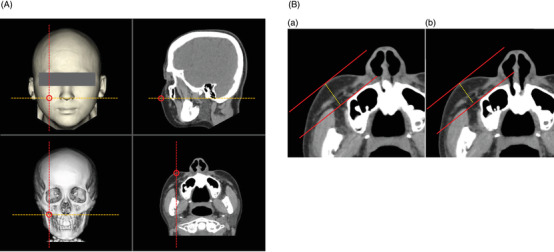
Measurements of the cheek thickness. (A) Reconstructed images on a workstation. On a workstation, the thickest point of the cheek, known as the malar top, was accurately determined using reconstructed sagittal and axial images. The malar top was double‐checked through observations from the anterior side of the face by using a three‐dimensional computed tomography (3DCT) image. On 3DCT images of the face and facial bone, a sagittal image is shown as a red dotted line, and an axial image is shown as an orange dotted line. Spiral CT imaging data have a coordinate axis in the imaging space. Thus, the same point can be precisely indicated on 3DCT images of the face and facial bone, as well as all multiplanar reformatted axial and sagittal images by utilizing the spiral CT coordinates. The centers of the red open circles indicate shared coordinates. (B) Measurements of the cheek thickness both pre‐ and post‐ facial massage. (a) A reconstructed axial image pre‐massage. First, a straight red line was drawn along the surface of the facial bone on a reconstructed axial image of the pre‐facial massage cheek. Next, a red line parallel to it was drawn tangent to the surface of the malar top. The distance from the facial bone to the surface of the malar top was defined as the cheek thickness of the pre‐facial massage. (b) Reconstructed axial image post‐massage. Finally, a reconstructed axial image of the post‐massage cheek at the same level as that used for the pre‐massage evaluation was created using the stationary facial bone as the basis for measurements, and the post‐massage cheek thickness was measured using the same method as that used for the pre‐facial massage measurement. Change rate of the cheek thickness (%)  = (cheek thickness post‐massage/cheek thickness pre‐massage) × 100

Cheek thickness measurements were performed on both cheeks (total 10 cheeks) of five participants (Figure 2). First, as shown in Figure 2A, the pre‐facial massage sagittal and axial images as well as 3DCT images were obtained on a workstation. Next, the thickest point of the cheek, known as the pre‐massage malar top, was accurately determined by combining the coordinates of the reconstructed sagittal and axial images, and then double‐checked through observations from the anterior side of the face using 3DCT images. On a reconstructed axial image, the distance from the facial bone to the surface of the malar top was defined as the thickness of the cheek. Finally, as shown in Figure 2B, a reconstructed axial image of the cheek at the same level as the pre‐massage image was created using the stationary facial bone as the landmark for measurements, and the post‐massage cheek thickness was measured.

Using these measurements, the change rate of the cheek thickness was calculated with the following formula:

Change rate of the cheek thickness (%) = (cheek thickness post‐massage/cheek thickness pre‐massage) × 100.

#### 2.3.2 Shifts in the location of the malar top

**FIGURE 3:**
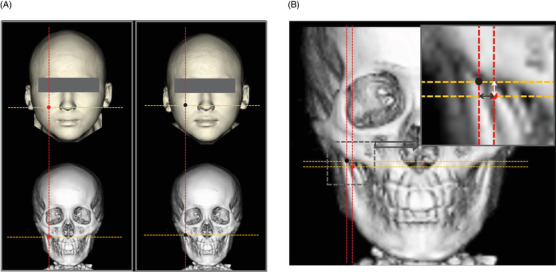
Measurements of the shift of the malar top due to facial massage. (A) Detection of the location of the malar tops both pre‐ and post‐massage. The malar tops of both checks pre‐ and post‐facial massage were accurately determined by a combination of reconstructed sagittal and axial images. The red circles on the 3DCT images of the face and facial bone indicated the malar tops pre‐massage, and the black circles on 3DCT images indicate the post‐massage malar tops. (B) Measurements of the difference in the malar tops pre‐ and post‐massage. Red and black circles with red and orange dotted lines are drawn on the facial bone. The differences in craniocaudal distance (white arrow in the right upper image) and horizontal distance were measured. The oblique distance by which the malar top shifted due to facial massage was calculated using the Pythagorean theorem from the craniocaudal and horizontal distances

Measurements of the massage‐induced differences in the location of the malar top were performed on 10 cheeks of five participants (Figure 3). As shown in Figure 3A, the malar tops of both cheeks were confirmed before and after the facial massage. As shown in Figure 3B, the facial massage‐induced differences in the malar top location were determined in terms of the craniocaudal and horizontal distances. The massage‐induced shift in the malar top was calculated using the Pythagorean theorem with the craniocaudal and horizontal distances.

#### 2.3.3 SMAS measurement

**FIGURE 4:**
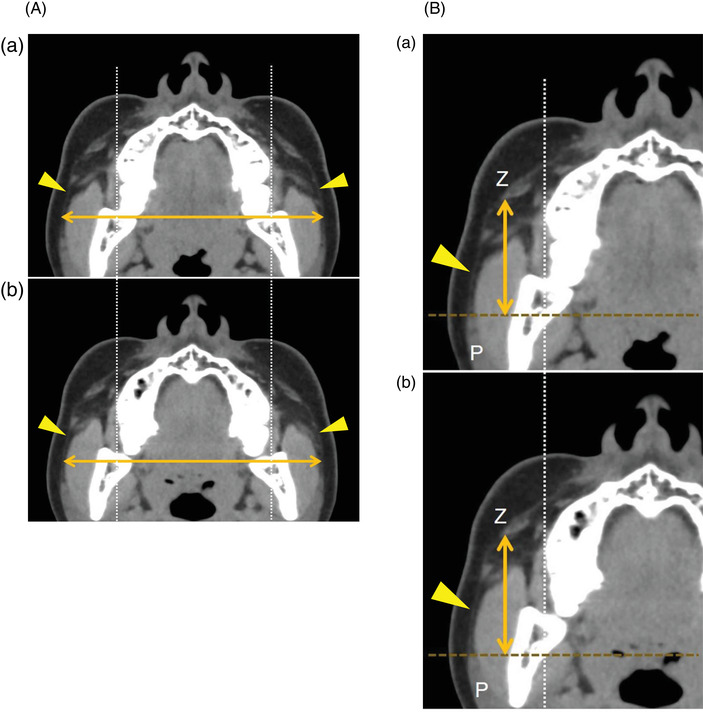
Superficial musculoaponeurotic system (SMAS) measurements. (A) Measurement of SMAS‐width. This computed tomography (CT) scan depicts the SMAS as a subcutaneous linear structure in the adipose tissue (yellow arrowheads). On a reconstructed axial CT image, the widest horizontal distance of the SMAS between the right and left margins of the SMAS was defined as the SMAS‐width. The SMAS‐width pre‐massage was measured. Subsequently, reconstructed axial post‐massage images corresponding to the same level as the pre‐massage image were created using the stationary facial bone as a basis for the measurements. The SMAS‐width post‐massage was then measured. Change rate of the SMAS‐width (%).  = (SMAS‐width post‐massage/SMAS‐width pre‐massage) × 100. (B) Measurement of SMAS‐height. On a reconstructed axial CT image, the highest vertical distance of the SMAS (yellow arrowhead) from the anterior edge of the parotid gland (P) to the back of the major zygomatic muscle (Z) was defined as the SMAS‐height. The SMAS‐height pre‐massage was measured, and the SMAS‐height post‐massage was measured on the reconstructed axial image. Change rate of the SMAS‐height (%). = (SMAS‐height post‐massage/SMAS‐height pre‐massage) × 100

As shown in Figure 4, the SMAS is the fascia sandwiched between the superficial and deep layers of the adipose tissue, and is recognized as a linear structure on CT.^7^, ^8^ On reconstructed axial CT images, the widest horizontal distance between the right and left margins of the SMAS was defined as the SMAS‐width (Figure 4A). The pre‐ and post‐massage SMAS‐widths were measured on the faces of the five participants. The highest vertical distance of the SMAS from the anterior edge of the parotid gland to the back of the major zygomatic muscle was defined as the SMAS‐height (Figure 4B), and the pre‐ and post‐massage SMAS‐heights of the 10 cheeks in the five participants were measured.

Using these data, the change rates of the SMAS‐width and the SMAS‐height caused by facial massage were calculated with the following formula:

Change rate of SMAS‐width (%)

 = (SMAS‐width post‐massage/SMAS‐width pre‐massage) × 100,

Change rate of the SMAS‐height (%)

 = (SMAS‐height post‐massage/SMAS‐height pre‐massage) × 100.

### 2.4 Statistical analysis

Participant age, cheek thickness, shifts of the malar top, SMAS‐width, and SMAS‐height are presented as mean ± standard deviation values. The mean values for cheek thickness, SMAS‐width, and SMAS‐height before and after massage were compared using the mean values of a paired *t*‐test.

A correlation analysis was performed to determine the relationship between the change rates of cheek thickness and SMAS‐width due to facial massage. Similarly, the correlation between the change rates of cheek thickness and the SMAS‐height due to facial massage was also determined.

Statistical analyses were performed using the StatMate V statistical software package (Nihon 3 B Scientific Inc., Niigata, Japan). Statistical significance was set at *P *< 0.05.

## 3 RESULTS

The overall quality of the 3DCT and reconstructed images of all participants was adequate and did not hinder measurements of the cheek thickness, location of the malar top, and the SMAS.

### 3.1 Cheek thickness

**FIGURE 5:**
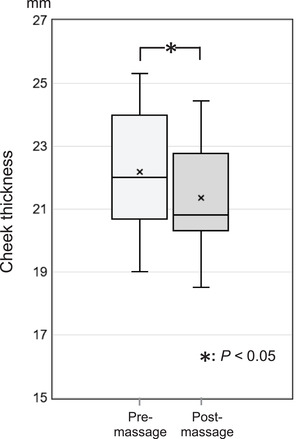
Changes in the cheek thickness due to facial massage. The mean value of cheek thickness (10 cheeks in 5 participants) pre‐ and post‐massage are 22.0 ± 1.34 mm and 21.2 ± 1.08 mm, respectively. Thus, the mean value of cheek thickness significantly decreased after facial massage (*P* < 0.05)

The mean pre‐massage thickness of the malar top was 22.0 ± 1.34 mm, and the cheek thickness decreased by 21.2 ± 1.08 mm due to facial massage. The mean change rate of cheek thickness was −0.8 ± 0.45%, and the mean cheek thickness value significantly decreased after facial massage (Figure 5, *P* < 0.05).

### 3.2 Shifts in the location of the malar top

**FIGURE 6:**
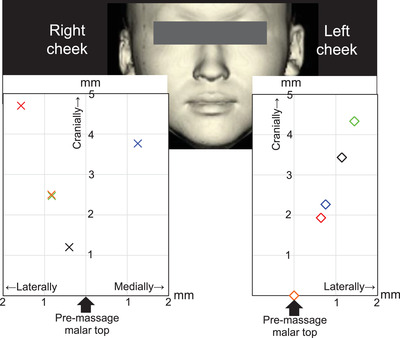
Shift in the location of the malar top due to facial massage. Individual differences in the massage effect were recognized, and there was a difference in the massage effect between the left and right sides even in one participant. They are as follows. Blue: participant 1, red: participant 2, black: participant 3, orange: participant 4, green: participant 5. On the right cheeks of the five participants, the malar tops shifted cranially toward. In assessments of horizontal shift, the malar tops shifted laterally and medially in four and one participant, respectively. On the other hand, the left cheeks of four participants showed cranial shifting of the malar top. In assessments of horizontal shift, the malar tops of four participants shifted laterally. The mean craniocaudal shift of the 10 malar tops was 2.7 ± 1.44 mm, with 9 malar tops shifting cranially and remaining at the same location. The mean horizontal shift of the 10 malar tops was 2.7 ± 1.48 mm, with eight malar tops shifting laterally, one shifting medially, and one remaining in the same location

As shown in Figure 6, the mean craniocaudal shift in the 10 malar tops of the five participants was 2.7 ± 1.44 mm, with nine malar tops shifting cranially and one remaining in the same location. The mean horizontal shift of the 10 malar tops was 2.7 ± 1.48 mm, with eight malar tops shifting laterally, one shifting medially, and one remaining in the same location. On the basis of the cranial and horizontal distances, the mean shifting distance of the malar top was calculated to be 3.9 ± 1.94 mm.

### 3.3 SMAS measurements and their relationships with cheek thickness

The mean pre‐ and post‐ massage SMAS‐widths of the five participants’ faces were 122.7 ± 4.35 mm and 122.4 ± 4.03 mm, respectively. The mean change rate of the SMAS‐width was −0.20% ± 1.01%. The SMAS‐width tended to decrease with massage; however, the difference was not statistically significant (*P *= 0.66).

The mean pre‐ and post‐massage SMAS‐heights of the 10 cheeks of the five participants were 37.8 ± 13.57 mm and 38.7 ± 13.75 mm, respectively. The mean change rate of the SMAS‐height was 2.6% ± 2.6%, and the mean SMAS‐height significantly increased after facial massage (*P* < 0.05).

**FIGURE 7:**
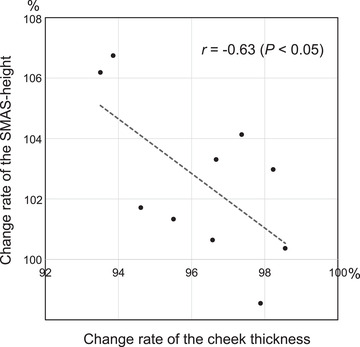
Correlation of the change rates of superficial musculoaponeurotic system (SMAS)‐height and cheek thickness. In the 10 cheeks of the five participants, a significant negative correlation was detected between the change rates of cheek thickness and SMAS‐height (*r* = −0.63; *P* < 0.05)

A positive correlation was observed between the change rates of cheek thickness and SMAS‐width; however, this correlation was not statistically significant (five faces of the five participants, *r* = 0.71; *P* = 0.18). A significant negative correlation was observed between the change rate of SMAS‐height and cheek thickness (Figure 7, 10 cheeks of the five participants, *r* = −0.63; *P* < 0.05).

## 4 DISCUSSION

Facial massage is empirically known to induce changes in the facial contours and improve facial sagging. However, one of the major problems in evaluating the effectiveness of facial massage is the difficulty in setting landmarks to evaluate the massage‐induced morphological changes of the face. We assume that objective evaluations of facial massage had not progressed for these reasons.

Therefore, we applied the breakthrough CT technique of spiral CT (for obtaining high‐resolution images) along with a workstation (for processing and analyzing a large amount of image) for the evaluation of beauty treatments. An objective quantify evaluation of facial massage was attempted.

In general, soft tissues, including the SMAS and subcutaneous adipose tissue, were shown to be susceptible to the effects of gravity.^10^, ^11^, ^12^ The gravity vector applied to the face is different in the upright and supine positions, and the morphology of the soft tissues is also different. Therefore, the facial appearance in a supine position is younger than those in standing or sitting positions.^10^, ^11^, ^13^, ^14^ Thus, evaluations of the facial state should be performed in a standing or sitting position. In our study, CT examination of the faces was performed in the coronal position, which corresponds to the standing position, as reported by Okuda et al.^11^

The workstation is installed with an application that uses various preset reconstruction algorithms, including dedicated face scan algorithms, to reconstruct 3DCT images of the face.^15^, ^16^ Volume‐rendering, based on an edge‐detection image processing system, was used in these 3D reconstructions. Spiral CT imaging data contain 3D information and include a coordinate axis for the area imaged using the spiral CT system. One of the advantages of the workstation is that the same point can be precisely indicated on 3DCT and all multiplanar reformatted axial and sagittal images by utilizing spiral CT coordinates. Moreover, in addition to the facial 3D state, the anatomical relationship between the facial appearance and the subcutaneous structures can be accurately displayed with this approach.^7^, ^8^, ^11^ A further innovation of our study was to use stationary facial bones, which were not changed by massage, as the landmarks for creating images for comparison before and after facial massage. This allowed us to quantify minute soft‐tissue changes caused by facial massage.

The malar tops of nine cheeks in five participants were shifted cranially by 2.7 ± 1.44 mm due to facial massage. Although the degree of the lifting effect varied, massage had a lifting effect on the cheeks in most of the participants. Subcutaneously, the SMAS‐height increased (from 37.8 ± 13.57 mm to 38.7 ± 13.75 mm, *P* < 0.05) and the pre‐massage cheek thickness decreased (from 22.0 ± 1.34 mm to 21.2 ± 1.08 mm, *P* < 0.05). We found a significant negative correlation between the change rates of the SMAS‐height and cheek thickness (*r* = −0.63; *P* < 0.05). These results suggest that the cranial shift of the soft tissues due to the lifting effect of facial massage contributed to a reduction in cheek thickness.

On the other hand, the SMAS‐width tended to decrease (from 122.7 ± 4.35 mm to 122.4 ± 4.03 mm) due to facial massage. Thus, the tightening effect of the massage may be related to the reduction of soft tissue. A positive correlation was inferred between the SMAS‐width and the rate of change in cheek thickness (*r* = 0.71; *P* = 0.18), although the sample size was small, and the differences were not statistically significant. The direction and change of the horizontal shift (medially or laterally) of the malar top due to facial massage differed among individuals. There was a difference in the massage effect between the left and right half of the face in each participant. Thus, individual differences were assumed to be present in the expression of the massage effect.

Increased blood and lymphatic flow are thought to be the main factors underlying the effectiveness of massages.^5^, ^17^, ^18^ In addition, Okuda et al. considered the involvement of the mobility of facial soft tissues, including the SMAS and subcutaneous adipose tissue.^13^ Thus, facial massage could have tightening and lifting effects, and these would contribute to morphological changes of the face, including the subcutaneous adipose tissue and the SMAS. The results of the present study support this hypothesis.

This study was a pilot study conducted prior to a large‐scale study that would enroll several participants. The possibility of quantifying the fine changes caused by facial massage was attempted. The limitations of this study were the small‐sample size, limited age range (29–36 years), and the factors that it does not take gender differences in the skin texture as well as the environment surrounding participants into consideration. However, despite the small number of participants, we were able to quantify the effectiveness of facial massage and conduct an objective analysis because the analysis using breakthrough CT technology is highly objective. We believe that the confirmation of the effects of massage, which had been discussed empirically, in an objective method is a highly significant finding. Future studies should aim to examine how factors such as age, gender differences, facial structures, and environment are involved in the effectiveness of facial massage in a larger number of subjects.

## 5 CONCLUSIONS

Using breakthrough CT technology, we conducted a detailed analysis of the effects of massage on the facial surface in this preliminary pilot study. Facial massage appeared to show lifting and tightening effects. It caused the cheeks to shift cranially, and thick cheeks became thinner. Moreover, the SMAS‐height increased. Our results provide useful information for beauty treatments and could contribute to the objective scientific literature for facial massages. We expect that these diagnostic imaging data for facial massage will facilitate the development of new techniques.

## CONFLICT OF INTEREST

The authors declared that there is no conflict of interest that could be perceived as prejudicing the impartiality of the research reported.
